# Chronic kidney disease-associated pruritus and patient-centred outcomes: a systematic review

**DOI:** 10.1007/s40620-025-02221-9

**Published:** 2025-02-25

**Authors:** Teng Wang, Jing Xin Goh, Shrey Seth, Linda Le Do, Wubshet Tesfaye, Kamal Sud, Connie Van, Fatima Small, Surjit Tarafdar, Ronald L. Castelino

**Affiliations:** 1 Sydney School of Pharmacy, Faculty of Medicine and Health, The University of Sydney, Sydney, NSW 2006 Australia; 2 School of Pharmacy, The University of Queensland, Brisbane, Australia; 3 Nepean Kidney Research Centre, Department of Renal Medicine, Nepean Hospital, Nepean and Blue Mountains Local Health District, Penrith, NSW Australia; 4 Sydney Medical School, Faculty of Medicine and Health, University of Sydney, Sydney, NSW Australia; 5 Pharmacy Department, Blacktown Hospital, WSLHD, Blacktown, NSW 2148 Australia; 6 Department of Renal Medicine, Blacktown Hospital, WSLHD, Blacktown, NSW Australia; 7 School of Medicine, Western Sydney University, Sydney, NSW Australia

**Keywords:** Chronic kidney disease, Pruritus, Systematic review, Outcomes

## Abstract

**Background:**

Chronic kidney disease-associated pruritus (CKD-aP) is a debilitating symptom that can significantly impact patients’ daily activities and quality of life. This systematic review aimed to assimilate the latest evidence on the relationship between CKD-associated pruritis and patient-centred outcomes.

**Methods:**

A comprehensive search was conducted to identify relevant studies in PubMed, Medline and Embase via OVID, CINAHL, and Web of Science from 2000 to June 2024. Quality appraisal and subsequent data extraction were performed using the Joanna Briggs Institute (JBI) tools and a modified extraction form derived from JBI.

**Results:**

The review included 29 studies with a total of 147,174 CKD patients, including those on haemodialysis (HD) and peritoneal dialysis (PD). The most frequently reported patient-centred outcomes included quality of life (*n* = 21), sleep quality (*n* = 17), anxiety/depression (*n* = 11) and mortality (*n* = 7). There was a paucity of data on patients in the pre-dialysis stages, those undergoing PD, and following a conservative (non-dialytic) pathway. The impact of CKD-associated pruritus on outcomes was contingent on the severity of CKD-associated pruritus. There was an association between increased medication usage, decreased compliance with HD treatments and higher rates of hospitalisation in patients experiencing severe pruritus.

**Conclusion:**

Our review underscores the pernicious impact of CKD-associated pruritus on patient outcomes and emphasises the importance of effective management to improve patient-centred outcomes. Additional investigations are warranted among patients undergoing PD, those in pre-dialysis stages, and on conservative (non-dialytic) pathways, to achieve a more comprehensive understanding of the impact of CKD-associated pruritus in these patient groups.

**Graphical abstract:**

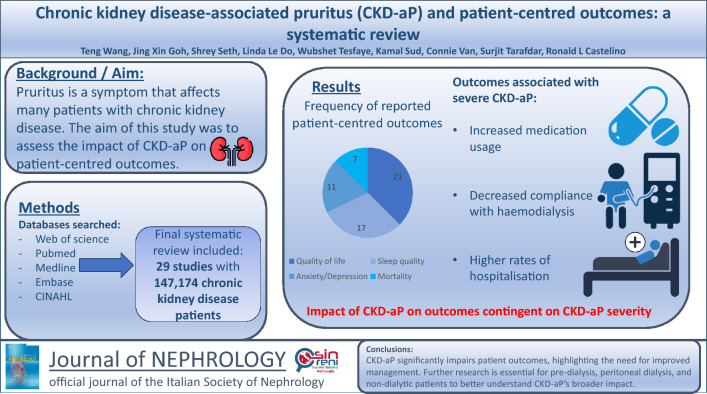

**Supplementary Information:**

The online version contains supplementary material available at 10.1007/s40620-025-02221-9.

## Introduction

Pruritus, a burdensome symptom associated with chronic kidney disease (CKD), is commonly referred to as chronic kidney disease-associated pruritus (CKD-aP) [1]. The prevalence of pruritus tends to increase with the severity of CKD and is notably more pronounced in individuals nearing or experiencing kidney failure [2, 3]. Current studies estimate the prevalence of CKD-associated pruritus to be as high as 90% among individuals undergoing dialysis [4]. Furthermore, the prevalence of CKD-associated pruritus and its associated complications are predicted to rise with the increasing incidence of CKD, owing to a concurrent rise in its risk factors, such as an ageing population, diabetes, hypertension and the resultant growing prevalence of patients requiring kidney replacement therapies [5–7].

Although multiple hypotheses have been proposed, the exact pathophysiological mechanism behind CKD-associated pruritus remains unknown. Four primary pathways have been identified, including disequilibrium in the activation of mu and kappa opioid receptors, central and peripheral neuropathy, disruption of the immune system accompanied by augmented concentration of interleukin-31, and accumulation of uraemic toxins in the subcutaneous tissue [8]. A multifactorial mechanism has been postulated that underlines complex interactions between dermal mast cells, nerve fibres, epidermal keratinocytes, and TH1 lymphocytes. CKD-associated pruritus has also been linked with heightened concentrations of β2-microglobin (a pruritogenic factor); upregulation of kappa-opioid receptors, which possess an antipruritic effect; and numerous cytokines secreted by TH1 lymphocytes, which may significantly contribute to the pathogenic pathways [9]. However, the central pathways involved in the development of CKD-associated pruritus and the definite pathogenic mechanism remain obscure.

Despite the high prevalence of CKD-associated pruritus, the evidence on optimal treatment and management strategies remains scarce [10]. Current pharmacological management for CKD-associated pruritus involves various topical and systemic therapies, including topical capsaicin, pramoxine, cromolyn sodium, gamma-linolenic acid, sericin, calcineurin inhibitors, systemic gabapentinoids, Mu-receptor antagonists, antihistamines, antidepressants, mast cell stabilisers and corticosteroids, all of which have variable efficacy and safety concerns [11–13]. However, there have been advancements in the treatment of CKD-associated pruritus, including the development of difelikefalin, a recently FDA-approved kappa opioid receptor agonist. It has demonstrated efficacy in reducing pruritus severity and improving quality of life in clinical studies, but further research is needed to better understand its impact on patient-centred outcomes [14, 15].

Given the high prevalence of this chronic condition and the absence of effective management, it is crucial to understand the impact of this often-debilitating symptom on patient-centred outcomes to optimise management strategies and therapeutic approaches. Therefore, the primary objective of this systematic review was to compile contemporary evidence to evaluate the impact of CKD-associated pruritus on patient-centred outcomes.

## Methods

This systematic review was conducted in accordance with the Preferred Reporting Items for Systematic Reviews and Meta-Analysis (PRISMA) guidelines [16].

### Evidence source

The search encompassed PubMed; Medline and Embase via OVID; CINAHL; and Web of Science, utilising relevant Medical Subject Headings (MeSH), Emtree, and CINAHL subject headings, along with specific keywords. Additional articles not initially identified were retrieved through reference lists and citation chaining. Search terms included “chronic kidney disease” AND “pruritus” and their analogues. Analogous terms for chronic kidney disease comprised renal disease, renal failure, kidney disease, kidney failure, dialysis, renal supportive care, kidney supportive care, and end stage kidney disease. Pruritus-related terms included itch* and chronic itch*.

### Study selection

Full-length articles in English, published since 2000, that focused on the impact of CKD-associated pruritus on patient outcomes were included. Criteria for inclusion were primary studies conducted in adults (18 years and over) and presenting quantitative data obtained using standardised instrumental scales. These scales included the Visual Analogue Scale [17], Skindex 29 [18], 5-D itch scale [19], Kidney Disease Quality of Life 36-item short form survey [20], Dermatology Life Quality Index [21], 12 or 36-Item Short Form Health Survey [22, 23], World Health Organization Quality-of-Life Scale [24], and ItchyQol [18]. The emphasis was on assessing CKD-associated pruritus severity and its consequent impact on patient-centred outcomes.

Conversely, studies conducted in the general population without CKD, paediatric or adolescent groups, and those solely published as abstracts were excluded. Additionally, studies utilising non-standardised scales for pruritus assessment or those with patient-centred outcomes but primarily focusing on interventions for pruritus management (for example, clinical trials for new treatments) were excluded.

Studies identified through the established search strategy underwent deduplication using both Endnote and Covidence software. Initial screening, which involved the assessment of article titles and abstracts, was conducted independently by one reviewer (TW) to determine the eligibility of the study. In cases of uncertainty, articles underwent full-text review. Discrepancies regarding eligibility prompted a secondary review and discussion with a second reviewer (WT) to reach a consensus.

### Outcomes

Outcomes assessed in the studies included patient-centred outcomes, such as quality of life, symptom scores, and sleep quality. Additionally, clinical outcomes such as psychosocial symptoms (including depression and anxiety), mortality, and hospitalisations were also included.

### Data extraction

Data extraction was independently performed by one reviewer (TW), including author, year, study design, population size, objective, country of origin, quantitative instrument used and outcomes. Relevant findings from the included studies were synthesised into a grid adapted from the Joanna Briggs Institute (JBI) extraction form.

### Study quality assessment

The methodological quality and bias of the acquired studies were assessed using the JBI critical appraisal tools [25]. For cross-sectional studies, a score of ≥ 7 indicated high quality, 4–7 denoted moderate-quality, and < 4 signified low-quality studies. In the case of case–control and cohort studies, a score of ≥ 10 was considered high quality, < 6 indicated low quality and scores in between were classified as moderate-quality studies.

## Results

**Fig. 1:**
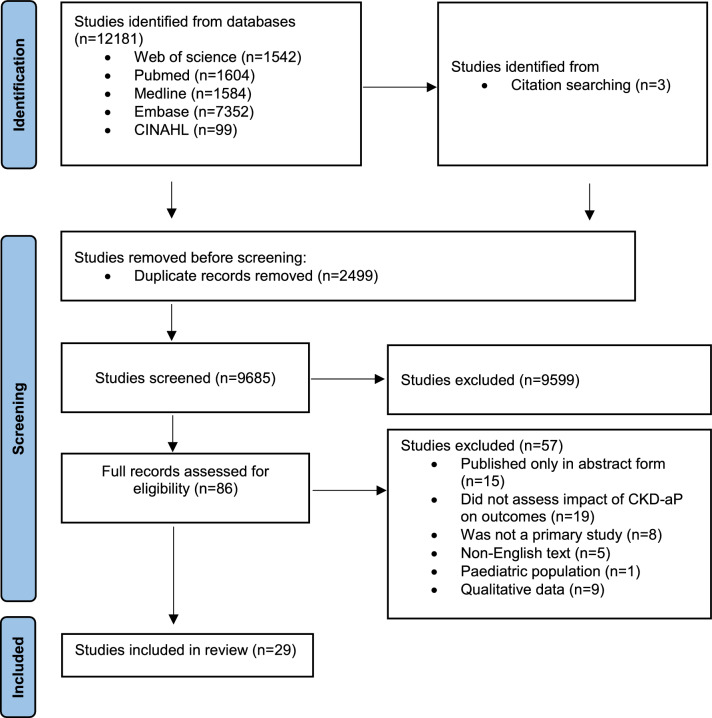
PRISMA flowchart illustrating the study selection process

A total of 12,181 studies were identified through the initial search. Following deduplication, 2499 articles were excluded. Subsequent title and abstract screening were performed on 9685 studies, with 9599 being deemed incongruent with the eligibility criteria and subsequently excluded. The remaining 86 studies underwent a full-text review, and 29 studies were included in the review. Figure 1 outlines the article selection process through a PRISMA chart.

### Characteristics of included studies

The included studies comprised 18 cross-sectional studies [26–43], 10 cohort studies [2, 3, 44–51], and 1 case–control study [52]. They were primarily conducted in Germany (*n* = 7) [3, 41, 42, 44, 46, 49, 50], followed by the United States (*n* = 6) [3, 38, 47–50], France (*n* = 5) [3, 38, 48–50], and Italy (*n* = 4) [3, 49–51]. The studies included a cumulative patient population of 147,174 individuals with CKD. Most studies focused on patients receiving dialysis, with 27 studies assessing the outcomes in HD patients [2, 3, 26–37, 39–47, 49–52], and only three studies investigating the outcomes in patients on both haemodialysis (HD) and peritoneal dialysis (PD) [40, 47, 51]. However, a much smaller number of studies included patients in either the pre-dialysis stage (*n* = 2) [38, 48] or both the dialysis and pre-dialysis stages (*n* = 1) [26]. Notably, all three studies included patients with advanced CKD on the conservative (non-dialysis) pathway. Among dialysis patients, the majority were undergoing HD (*n* = 67,479), while a small proportion were either on PD (*n* = 387) or in the pre-dialysis stage (*n* = 8296). One study included a total of 71,012 patients on dialysis but did not provide a breakdown between HD and PD [47].

The systematic review included a diverse range of studies employing various instruments to assess different aspects of patient-centred outcomes and overall well-being. Itch and its severity were comprehensively evaluated through different instruments, including the Visual Analogue Scale (*n* = 6) [2, 26, 39, 44, 46, 51], 5-D Itch Scale (*n* = 10) [27–29, 31–36, 43], Numerical Rating Scale (*n* = 1) [52], and 12-point Pruritus Severity Scale (*n* = 1) [37].

### Quality assessment of included studies

Using the range of risk JBI resources for assessing risk of bias of cross-sectional, cohort, and case–control studies, 5 studies were rated as high quality [30, 40, 48, 50, 51], while 24 studies were categorised as moderate quality [2, 3, 26–29, 31–39, 41–47, 49, 52] (Online Resource 1).

### Association between CKD-associated pruritus and patient-centred outcomes

Several studies examined the correlation between CKD-associated pruritus and various patient-centred outcomes, such as quality of life, sleep quality, and clinical outcomes such as, anxiety, depression, hospitalisation, and mortality. Majority of the studies focussed on the association between CKD-associated pruritus and quality of life, which was explored in 21 studies [3, 26, 29, 31–33, 36–43, 45–47, 49–52], closely followed by sleep quality (*n* = 17) [2, 3, 27, 28, 30, 34, 35, 37, 38, 40–42, 45, 46, 49], and anxiety/depression (*n* = 11) [3, 30, 36, 38, 39, 41, 42, 46, 49, 50]. However, a relatively smaller proportion of studies assessed the link between CKD-associated pruritus and other clinical outcomes, such as mortality (*n* = 7) [2, 3, 44, 45, 48–50] and hospitalisation (*n* = 3) [48–50] (Table 1).

**Table 1:** Characteristics of included studies

Author, year	Study design	Population size	Objective	Country of origin	Instrument used	Outcomes assessed	Results/findings
Adejumo et al. 2016 [24]	Cross-sectional study	69 pre-dialysis patients and 22 haemodialysis (HD) patients	Examine the association between chronic kidney disease-associated pruritus (CKD-aP) and quality of life	Nigeria	Skindex-10 Visual Analogue Scale	Quality of life	Significant positive association was observed between CKD-aP severity and quality of life (*p* = < 0.001, *r* = 0.56)
Aybek et al. 2022 [25]	Cross-sectional study	219 HD patients	Evaluate impact of CKD-aP on quality of sleep	Turkey	5-D itch scale Pittsburgh Sleep quality index	Sleep quality	A positive and moderately significant correlation was identified between pruritus severity and sleep quality (*r* = 0.509, * p* = 0.002)
Daraghmeh et al. 2022 [26]	Cross-sectional study	250 HD patients	Investigate the impact of CKD-aP on the quality of sleep	Palestine	5-D itch Scale Pittsburgh Sleep quality index	Sleep quality	A significant correlation was observed between CKD-aP severity and sleep quality (*r* = 0.235, * p* > 0.001)
Grochulska et al. 2019 [42]	Cohort study	724 HD patients	To report the mortality rate among HD patients with and without CKD-aP	Germany	Visual Analogue Scale	Mortality	No significant difference in survival rates was found between patients with and without pruritus. Mortality initially linked to moderate to severe itch (RR = 1.2; * p* < 0.02) but lost significance after adjusting for age and sex
Ibrahim et al. 2016 [50]	Case–control study	100 maintenance HD patients with pruritus and 100 HD patients without pruritus	Assess the quality of life in patients with and without CKD-aP	Egypt	Numerical Rating Scale World Health Organization Quality of Life Brief Version	Quality of life	The quality of life for HD patients with pruritus was significantly impaired (*p* < 0.05) compared to those without pruritus
Kimata et al. 2014 [43]	Observational cohort study	6480 HD patients	Examine the correlation between CKD-aP and quality of life, sleep quality, medication use, and mortality	Japan	36-Item Short Form Survey 12-Item Short Form Survey	Sleep quality Quality of life Mortality	Patients with moderate to extreme pruritus were more likely to experience fatigue (adjusted odds ratio = 2.2–5.8, * p* < 0.0001), poor sleep quality (adjusted odds ratio = 1.9–3.7, * p* < 0.0001), lower quality of life adjusted odds ratio = 2.3–6.7 points (*p* < 0.0001), and higher odds of taking antihistamines, antidepressants, sedatives and benzodiazepines compared to those with no/mild pruritus Additionally, pruritus in HD patients was associated with a 23% higher mortality risk
Kurniawan et al. 2022 [27]	Cross-sectional study	39 HD patients	Investigate the effect of pruritus on the quality of life in CKD patients undergoing HD	Indonesia	5-D itch scale Dermatology Life Quality Index	Quality of life	Statistically significant positive correlation was observed between the severity of CKD-aP and the quality of life
Lopes et al. 2012 [28]	Cross-sectional study	980 HD patients	Investigate depressive symptoms and assess sleep quality in HD patients with CKD-aP	Brazil	Kidney Disease Quality of Life 36-Item Short Form Centre for Epidemiological Studies Depression	Sleep quality Depression	The severity of CKD-aP was associated with poorer sleep quality (*p* < 0.001) and greater depressive symptoms (*p* < 0.001)
Mollaoğlu et al. 2021 [29]	Descriptive correlational and cross-sectional study	67 HD patients and 37 non-HD patients	Investigate pruritus and dermatological quality in CKD patients who receive or do not receive renal replacement therapy	Turkey	5-D Itch scale Dermatology Life quality index	Quality of life	As pruritus increased, the rate of adversely affecting the dermatological quality of life increased
Narita et al. 2006 [2]	Cohort study	1773 HD patients	Investigate the impact of CKD-aP on sleep quality and its prognostic significance for mortality in patients undergoing HD	Japan	Visual Analogue Scale [Own sleep scale]	Sleep quality Mortality	The severity of CKD-aP correlated with the frequency of sleep disturbances (*p* < 0.0001) and application of anti-pruritic treatment including antipruritic lotions, antihistamines and sedatives (*p* < 0.0001). Additionally, the severity of CKD-aP was an independent predictive factor for mortality
Pisoni et al. 2006 [3]	Cohort study	18,801 HD patients	Investigate the relationship of CKD-aP with quality of life, sleep quality, depression, medication use and mortality	Australia, Belgium, Canada, France, Germany, Italy, New Zealand, Spain, Sweden, United Kingdom and United States of America	36-Item Short Form Survey 12-Item Short Form Survey	Sleep quality Quality of life Mortality Depression	Patients experiencing moderate to extreme pruritus were more likely to report feelings of fatigue (adjusted odds ratio = 2.3–5.2, * p* < 0.0001) and exhibit poor sleep quality (AOR = 1.9–4.1, * p* ≤ 0.0002). They were also more likely to have physician-diagnosed depression (AOR = 1.3–1.7, * p* ≤ 0.004) and lower quality of life scores, 3.1–8.6 points lower (*p* < 0.0001) compared to patients with no or mild pruritus. Pruritus in HD patients was associated with a 17% higher mortality risk (*p* < 0.0001), although this association lost significance after adjusting for sleep quality measures
Plewig et al. 2019 [44]	Cohort study	104 HD patients	Assess the impact of CKD-aP on patients' quality of life after 4 years	Germany	12-Item Short Form Survey Hospital Anxiety and Depression Scale Visual Analogue Scale ItchyQoL	Quality of life Anxiety Sleep quality	Participants with persistent CKD-aP reported suffering from higher severity of itch, poor quality of life, greater anxiety, and a reduced sleeping time Osteoporosis impacted participants with persistent CKD-aP more frequently compared to patients without ongoing pruritus (*p* = 0.03)
Ramakrishnan et al. 2014 [45]	Cohort study	71,012 HD and peritoneal dialysis (PD) patients—preliminary analysis 38,315 HD patients—detailed analysis	To investigate the impact of CKD-aP on quality of life, medication use and HD compliance	United States of America	Kidney Disease Quality of Life 12-Item Short Form Kidney Disease Quality of Life 36-Item Short Form	Quality of life Medication use HD compliance	CKD-aP severity is correlated with poor quality of life, increased intravenous antibiotic usage (*p* < 0.0001) and antipruritic agents, higher doses of iron (*p* < 0.0001) and erythropoiesis-stimulating agents (*p* < 0.0001) and decreased compliance with HD treatment
Rehman et al. 2018 [33]	Cross-sectional study	354 HD patients	Investigate the association between CKD-aP and sleep quality in patients undergoing HD	Pakistan	5-D itch scale Pittsburgh sleep quality index	Sleep quality	The severity of CKD-aP was significantly associated sleep quality (*p* < 0.001)
Rehman et al. 2019 [32]	Cross-sectional study	334 HD patients	To investigate the impact of CKD-aP on sleep quality of patients undergoing HD	Malaysia	Malay 5-D itch scale Malay Pittsburgh Sleep Quality Index	Sleep quality	The severity of CKD-aP was significantly associated with sleep quality (*p* < 0.001)
Rehman et al. 2019 [30]	Cross-sectional study	354 HD patients	Investigate the association between CKD-aP and the quality of life patients undergoing HD	Pakistan	Urdu 5-D itch scale Functional Assessment of Non-Life Threatening Conditions	Quality of life	The severity of CKD-aP was significantly associated with quality of life (*p* < 0.001)
Rehman et al. 2020 [31]	Cross-sectional study	334 HD patients	Investigate the association between CKD-aP and quality of life of patients undergoing HD	Malaysia	Malay 5-D itch scale Malay Functional Assessment of Non-Life Threatening Conditions	Quality of life	The severity of CKD-aP was significantly associated with quality of life (*p* ≤ 0.001)
Satti et al. 2019 [34]	Cross-sectional study	173 male HD patients	Investigate the impact of CKD-aP on quality of life	Pakistan	5-D itch scale Dermatology Life Quality Index Patient Health Questionnaire-9	Quality of life Depression	CKD-aP severity is correlated with depressive symptoms and poor quality of life (*p* < 0.05)
Scherer et al. 2023 [46]	Longitudinal cohort study	4410 non-dialysis CKD patients	Investigate the association with CKD-aP and CKD progression, kidney replacement therapy initiation, mortality, hospitalisation, cardiovascular events, infection events	Brazil, France, United States of America	Kidney Disease Quality of Life 36-item survey	Mortality Hospitalisation	Severity of pruritus was associated with higher rates of all-cause hospitalisation (*p* < 0.001), cardiovascular events (p 0.04) and infection events (*p* = 0.04) but no association was found with renal replacement therapy initiation (*p* = 0.20) or CKD progression (*p* = 0.87) Compared to patients who reported being not at all bothered by itchy skin, patients who were extremely bothered had a higher rate of all-cause mortality (HR, 1.74; 95% CI, 1.11–2.73), all-cause hospitalisation (HR, 1.56; 95% CI 1.11–2.18), and CV events
Shetty et al. 2023 [35]	Cross-sectional observational study	120 HD patients	Determine the impact of CKD-aP on health-related quality of life and sleep in HD patients	India	12-Point pruritus severity scale Skindex-10 Itch Medical Outcomes Study	Quality of life Sleep quality	Quality of sleep is significantly dependent on severity of pruritus (*p* < 0.001) Patients with increased pruritus severity demonstrated significantly worse quality of life (*p* < 0.001)
Sukul et al. 2019 [36]	Cross-sectional study	3780 patients with stages 3–5 CKD not on dialysis	Investigate CKD-aP associations with patient-reported outcomes	United States of America, France, and Brazil	Kidney Disease Quality of Life 36-Item Short Form Centre for Epidemiologic Studies Depression scale	Quality of life Depression Sleep quality	Patients with CKD-aP experienced lower quality of life, a higher prevalence of depression, and more restless sleep compared to those without CKD-aP. These negative outcomes worsened with increasing severity of CKD-aP
Sukul et al. 2021 [47]	Cohort study	23,264 HD patients	Investigate the association between CKD-aP and patient outcomes in patients undergoing HD	Australia, New Zealand, Belgium, Canada, China, France, Bahrain, Kuwait, Oman, Qatar, Saudi Arabia, United Arab Emirates, Germany, Italy, Japan, Russia, Spain, Sweden, Turkey, United Kingdom, and United States of America	Kidney Disease Quality of Life 36-Item Short Form Centre for Epidemiological Studies Depression	Mortality Hospitalisation Quality of life Depression Sleep quality	Patients with severe CKD-aP faced higher mortality rates (all-cause, cardiovascular, and infection-related) and increased hospitalisations. Those significantly bothered by CKD-aP also had a higher rate of hospitalization for mental status changes/confusion. Extreme CKD-aP severity was linked to a higher likelihood of withdrawing or skipping dialysis, unemployment, and longer recovery times after dialysis. There was a monotonic association between CKD-aP severity and self-reported recovery time. Additionally, CKD-aP severity correlated strongly with lower health-related quality of life (HRQOL), depressive osteoymptoms, poor sleep quality, and experiences of dizziness and lethargy
Sukul et al. 2023 [48]	Prospective cohort study	7976 HD patients	Assess patient reported outcomes of those with and without CKD-aP over a 1 year period	Australia, New Zealand, Belgium, Canada, China, France, Bahrain, Kuwait, Oman, Qatar, Saudi Arabia, United Arab Emirates, Germany, Italy, Japan, Russia, Spain, Sweden, Turkey, United Kingdom, and United States of America	Kidney Disease Quality of Life 36-item short form survey Center for Epidemiological Studies Depression	Depression Quality of life Mortality Hospitalisation Sleep quality	Strong association between change in pruritus symptoms and change in patient reported outcomes such as sleep quality and depressive symptoms (*p* < 0.05) Patients with CKD-aP had poorer quality of life as compared to those without CKD-aP Patients with CKD-aP had higher rates of all-cause mortality, all-cause hospitalisation and cardiovascular events compared to those without CKD-aP
Susel et al. 2014 [37]	Cross-sectional study	200 HD patients	Evaluate the impact of CKD-aP on quality of life and depressive symptoms in patients with end-stage kidney disease	Poland	Visual Analogue Scale Dermatology Life Quality Index 36-Item Short Form Survey Beck’s depression inventory	Quality of life Depression	Severity of CKD-aP was significantly associated with poor quality of life and depression (*p* < 0.0001)
Tessari et al. 2009 [49]	Cohort study	139 HD and 30 PD patients	Investigate the impact of CKD-aP on patients’ quality of life	Italy	Skindex-29 Visual Analogue Scale 36-Item Short Form Survey	Quality of life Sleep quality	The presence and severity of CKD-aP had no association with quality of life physical (*p* = 0.60) and mental scores (*p* = 0.49) CKD-aP was a predictor of poor sleep (odds ratio 8.4, * p* < 0.0001) Patients with pruritus reported poor sleep (59%) compared to patients without pruritus (11%) (*p* < 0.001)
Van der willik et al. 2022 [38]	Cross-sectional study	2583 HD patients and 357 PD patients	Investigate impact of CKD-aP on quality of life, sleep and psychological symptoms in dialysis patients	Netherlands	Dialysis symptom index 12-Item Short Form Survey	Quality of life Sleep quality Psychological symptoms	Patients with CKD-aP experienced a lower physical (*p* < 0.001) and mental (*p* < 0.001) HRQOL compared to those without CKD-aP There was no notable correlation between itchiness and sleep issues (*p* = 0.52 and * p* = 0.22, respectively) or CKD-aP and psychological symptoms (*p* = 0.66 and * p* = 0.29, respectively)
Weiss et al. 2015 [39]	Cross-sectional study	860 HD patients	Investigate patient outcomes associated with CKD-aP	Germany	12-Item Short Form Survey Hospital and Anxiety Depression Scale Itch-related QOL	Quality of life Anxiety Depression Sleep quality	The presence of CKD-aP was found to have no association with anxiety and depression but the severity was significantly associated with anxiety but not depression Patients with greater CKD-aP severity showed greater impairment in itch related quality of life however was not statistically significant (*p* = 0.50) Impaired sleep quality was significantly associated with the presence of CKD-aP but not its severity
Weiss et al. 2016 [40]	Cross-sectional study	860 HD patients	Investigate the health-related quality of life in CKD-aP patients	Germany	12-Item Short Form Survey Hospital and anxiety depression scale ItchyQol	Quality of life Anxiety Depression Sleep quality	Itch-specific HRQOL (*p* < 0.005) and anxiety worsened with increasing severity of CKD-aP but not for depression No association identified between severity of CKD-aP and sleep quality
Xie et al. 2022 [41]	Cross-sectional study	269 HD patients	Investigate the impact of CKD-aP on quality of life in patients undergoing HD	China	12-Item Short Form Survey 5-D Itch Scale	Quality of life	CKD-aP was significantly associated with physical and mental component summary (*p* < 0.001) and (*p* < 0.001)

#### Quality of life

Quality of life was measured using a range of tools, with seven studies utilising the 12-Item Short Form Survey [3, 40–43, 45, 46], six studies implementing the Kidney Disease Quality of Life 36-Item Short Form [30, 38, 47–50], four studies using the Dermatology Life Quality Index [29, 31, 36, 39], two studies applying the Functional Assessment of Non-Life-Threatening Conditions [32, 33] and Skindex-10 [26, 37], with one study each using the Skindex-29 [51], Kidney Disease Quality of Life 12-Item Short Form [47] and the World Health Organization Quality of Life Brief Version [52]. Two studies used the ItchyQoL to assess itch-specific quality of life [42, 46]. Among the 21 studies assessing quality of life [3, 26, 29, 31–33, 36–43, 45–47, 49–52], eight studies revealed a significant impact of CKD-associated pruritus on the outcomes [3, 38, 40, 41, 45, 46, 51, 52]. Furthermore, 11 studies indicated that the extent of this impact was closely associated with the severity of itching [26, 29, 32, 33, 36, 38, 39, 42, 47, 49, 50] (Table 2).

**Table 2:** Summary of key outcome findings from each quantitative study

Author(s)	Quality of life	Sleep quality	Psychosocial symptoms	Mortality
Adejumo et al. 2016 [24]	+			
Aybek et al. 2022 [25]		+		
Daraghmeh et al. 2022 [26]		+		
Grochulska et al. 2019 [42]				✓^−^
Ibrahim et al. 2016 [50]	X			
Kimata et al. 2014 [43]	X	X		✓
Kurniawan et al. 2022 [27]	+			
Lopes et al. 2012 [28]		+	+	
Mollaoğlu et al. 2021 [29]	✓			
Narita et al. 2006 [2]		+		✓
Pisoni et al. 2006 [3]	X	X	X	✓^−^
Plewig et al. 2019 [44]	X	X	X	
Ramakrishnan et al. 2014 [45]	+			
Rehman et al. 2018 [33]		+		
Rehman et al. 2019 [32]		+		
Rehman et al. 2019 [30]	+			
Rehman et al. 2020 [31]	+			
Satti et al. 2019 [34]	+		+	
Scherer et al. 2023 [46]				+
Shetty et al. 2023 [35]	+	+		
Sukul et al. 2019 [36]	+ X	+ X	+ X	
Sukul et al. 2021 [47]	+	+	+	+
Sukul et al. 2023 [48]	X	X	X	X
Susel et al. 2014 [37]	+		+	
Tessari et al. 2009 [49]	X ← →	✓		
Van der willik et al. 2022 [38]	X	←	←	
Weiss et al. 2015 [39]	X^−^	✓ →	← +*	
Weiss et al. 2016 [40]	+	→	+*	
Xie et al. 2022 [41]	✓			

Blank cells indicate outcome was not assessed by study

+ Significant association with severity of itching and impact on outcome

✓ Itching is associated with clinical outcome

X Itching has significant impact on outcome

^−^Not statistically significant

← No correlation between itching and outcome

→ No correlation between severity of itching and outcome

^*^No significant correlation with depression

#### Sleep outcomes

Assessment of sleep quality was predominantly conducted by either using a self-developed scale or sections of another scale like the Center for Epidemiological Studies–Depression questionnaire (*n* = 9)[2, 3, 38, 41, 42, 45, 46, 49, 50], while the Pittsburgh Sleep Quality Index was employed in four studies [27, 28, 34, 35]. In the analysis of 17 studies exploring the impact of pruritus on sleep [2, 3, 27, 28, 30, 34, 35, 37, 38, 40–42, 45, 46, 49–51], five studies identified a significant negative effect on sleep quality [3, 38, 45, 46, 50]. Additionally, nine studies demonstrated a notable association between the severity of itching and its impact on sleep outcomes [2, 27, 28, 30, 34, 35, 37, 38, 49]. However, two studies reported no discernible correlation between the severity of pruritus and sleep quality [41, 42] (Table 2).

#### Other outcomes

Anxiety and depression were evaluated through the Hospital Anxiety and Depression Scale in three studies [41, 42, 46], while depression alone was assessed using the Centre for Epidemiological Studies Depression scale in four studies [30, 38, 49, 50], and Beck’s depression inventory [39], 12- or 36-Item Short Form Health Survey [3] and the Patient Health Questionnaire-9 [36] were used in one study each. However, one study did not distinguish the psychological symptoms and reported no association with itching [40]. Of the 11 studies examining the impact of pruritus on depression and anxiety [3, 30, 36, 38–42, 46, 49, 50], 3 studies reported a significant effect of CKD-associated pruritus on psychosocial symptoms [3, 38, 46]. Moreover, seven studies highlighted a correlation between the impact on depression/anxiety and the severity of pruritus [30, 36, 38, 39, 41, 42, 49] (Table 2).

Seven studies reported associations between CKD-associated pruritus and mortality [3, 44, 45, 48–50, 53]. Two studies reported a significant correlation [48, 49] and one study reported a notable correlation between them.[50]. Nonetheless, statistical significance was not maintained in two of these studies after adjusting for patient factors and sleep quality [3, 44]. One study reported a significant correlation between severity of itching and higher rates of hospitalisation due to infections, mental, and cardiovascular conditions [49]. Severe pruritus was also linked with unemployment and longer post-dialysis recovery time [49]. Notably, one study compared the severity of pruritus among various dialysis modalities and revealed that PD patients experienced milder pruritus as compared to patients undergoing HD [54].

## Discussion

Our study presents a comprehensive systematic review exploring the impact of CKD-associated pruritus on a range of patient outcomes. The existence of CKD-associated pruritus is associated with detrimental effects on various patient-centred outcomes. Notably, it impacts quality of life, sleep quality, and psychosocial well-being and may contribute to symptoms of depression and anxiety. Additionally, CKD-associated pruritus is linked to an elevated risk of hospitalisation and mortality. The severity of CKD-associated pruritus also emerges as a crucial determinant, influencing the magnitude of adverse outcomes including quality of life, sleep quality and psychosocial well-being.

Studies have postulated that the impact of CKD-associated pruritus on both quality of life and mortality may be associated with its effect on poor sleep quality. This hypothesis finds support in a large-scale study where the association between CKD-associated pruritus, quality of life and mortality was significantly diminished after adjusting for sleep quality [3]. Hence, addressing and alleviating poor sleep quality could be an advantageous approach in managing the adverse implications of CKD-associated pruritus and potentially mitigating its impact on quality of life and mortality. Despite these positive signals, there is a paucity of evidence in patients undergoing PD and those receiving conservative management for advanced CKD. Future research should emphasise on bridging this gap in the literature and build the evidence pool in these scarcely investigated cohorts. CKD-associated pruritus is also implicated in an increased healthcare burden with its presence associated with heightened usage of intravenous antibiotics and higher doses of erythropoiesis-stimulating agents and iron[47]. Furthermore, individuals with CKD-associated pruritus exhibit reduced compliance with dialysis treatments, underscoring the broader impact on health and treatment adherence in patients with CKD-associated pruritus [47].

Pruritus is significantly pronounced in patients with kidney failure, with a prevalence of up to 90% in individuals undergoing dialysis [3, 53]. Dialysis has demonstrated efficacy in reducing the severity of CKD-associated pruritus due to the removal of uraemic toxins [55]. On comparing the severity of CKD-associated pruritus across dialysis modalities, one study found that patients on PD exhibited less severe pruritus than those undergoing HD [54]. This may be attributed to factors such as, daily dialysis sessions for PD and a trend of greater residual kidney function in PD patients [54, 55]. However, the current understanding of the relationship between dialysis modality and CKD-associated pruritus remains unclear and may be multifactorial. Notably, the application of a high-flux dialyser in HD has demonstrated effectiveness in alleviating pruritus symptoms [56, 57].

Considering the impact of CKD-associated pruritus outcomes is contingent on severity, it may suggest that patients with CKD-associated pruritus undergoing HD may experience a more pronounced impact. Despite this implication, there is a dearth of evidence directly comparing outcomes between HD and PD in the context of CKD-associated pruritus. Recent studies comparing outcomes across dialysis modalities indicated that PD demonstrated non-inferior or superior quality of life outcomes in comparison to HD, including overall quality of life, physical functioning, burden of kidney disease [58], and lifestyle flexibility [59]. Moreover, other studies have reported similar mortality rates between HD and PD in large-scale analyses [60–62]. Overall, the discrepancies highlight the complexity between dialysis modalities and CKD-associated pruritus outcomes. Further studies investigating the impact of CKD-associated pruritus on outcomes in PD are warranted to gain a thorough understanding and to compare outcomes between dialysis modalities, which may serve as potential treatment avenues to improve outcomes in those afflicted by CKD-associated pruritus.

Given that the prevalence of CKD-associated pruritus increases with the progression of CKD, it may contribute to further worsening of patient outcomes. However, a notable gap exists in the literature concerning studies that specifically investigate outcomes in individuals across CKD stages, especially those with advanced CKD who have not yet initiated dialysis and those on a conservative (non-dialytic) pathway. Nevertheless, one study incorporated in this review reported the presence of moderate to extreme pruritus in up to 24% of patients across all CKD stages [38]. Notably, this cohort exhibited a higher prevalence of moderate to extreme xerosis and restless sleep compared to those without pruritus [38].

The knowledge gap is also particularly relevant to patients on PD and those receiving kidney supportive care [63], where management of symptoms may be more effective as compared to patients not receiving kidney supportive care. This underscores the necessity for additional studies on CKD-associated pruritus across diverse populations to gain a comprehensive understanding of outcomes beyond those undergoing dialysis.

This review is not without its limitations. Methodologically, the impact of CKD-associated pruritus on outcomes in many studies was often reported as a secondary outcome. Consequently, if a study failed to explicitly mention the key words in the title or abstract, there was a possibility of exclusion during the screening process. The included studies also present limitations. Many of them are cross-sectional studies, and there is a case–control study, which hinders the establishment of causal relationships between CKD-associated pruritus and outcomes. As such, the associations identified between CKD-associated pruritus and outcomes should be interpreted with caution. Furthermore, a considerable number of studies were conducted predominantly in HD populations, small sample sizes, or a single location, thereby limiting the generalisability of the findings to other population groups, including those undergoing PD, in the pre-dialysis stage, or receiving kidney supportive care. Additionally, there was a notable dearth of pruritus or kidney-specific instruments utilised in the studies, and a majority of them were of moderate quality. This reduces the validity of the measured exposure and outcomes, highlighting the need for more rigorous and standardised approaches in future research on CKD-associated pruritus.

Overall, this systematic review underscores the adverse consequences of CKD-associated pruritus and emphasises the impact of its severity on both patient-centred and clinical outcomes of CKD-associated pruritus. The substantiated effects of CKD-associated pruritus on quality of life and sleep quality, especially in the HD population, highlight the significance of addressing this condition. However, the need for additional studies remains evident, particularly concerning outcomes in various dialysis modalities and diverse populations where comprehension is limited. Further research in these areas is crucial to enhance our understanding of the comprehensive impact of CKD-associated pruritus and inform effective management strategies.

## Supplementary Information

Below is the link to the electronic supplementary material.

Supplementary file1 (DOCX 45 KB)

## Data Availability

The data used during the study are available on reasonable request.
