# Supplementary material for: Chronic kidney disease-associated pruritus and patient-centred outcomes: a systematic review
Source: J Nephrol. 2025 Feb 25;38(2):371–91. doi: 10.1007/s40620-025-02221-9 (PMC11961476; doi:10.1007/s40620-025-02221-9)
Supplement: Supplementary file 1 — Supplementary file1 (DOCX 45 KB) [file 40620_2025_2221_MOESM1_ESM.docx]

**A1.** Appraisal of methodological quality of included cross-sectional studies

| Citation | Q1 | Q2 | Q3 | Q4 | Q5 | Q6 | Q7 | Q8 |
| --- | --- | --- | --- | --- | --- | --- | --- | --- |
| Adejumo et al. 2016 [24] | Y | Y | Y | Y | N | N | Y | Y |
| Aybek et al. 2022 [25] | Y | Y | Y | Y | N | N | Y | Y |
| Daraghmeh et al. 2022 [26] | Y | Y | Y | Y | N | N | Y | Y |
| Kurniawan et al. 2022 [27] | Y | Y | Y | Y | N | N | Y | Y |
| Lopes et al. 2012 [28] | Y | Y | Y | Y | Y | Y | Y | Y |
| Mollaoglu et al. 2021 [29] | Y | Y | Y | Y | N | N | Y | Y |
| Rehman et al. 2018 [33] | Y | Y | Y | Y | N | N | Y | Y |
| Rehman et al. 2019[32] | Y | Y | Y | Y | N | N | Y | Y |
| Rehman et al. 2019 [30] | Y | Y | Y | Y | N | N | Y | Y |
| Rehman et al. 2020 [31] | Y | Y | Y | Y | N | N | Y | Y |
| Satti et al. 2019 [34] | Y | Y | Y | Y | N | N | Y | Y |
| Shetty et al. 2023 [35] | Y | Y | Y | Y | N | N | Y | Y |
| Sukul et al. 2019 [36] | Y | Y | Y | Y | N | N | Y | Y |
| Susel et al. 2014 [37] | Y | Y | Y | Y | N | N | Y | Y |
| Tessari et al. 2009 [49] | Y | Y | Y | Y | Y | N | Y | Y |
| Van der willik et al. 2022 [31] | Y | Y | Y | Y | Y | Y | Y | Y |
| Weiss et al. 2015 [39] | Y | Y | Y | Y | N | N | Y | Y |
| Weiss et al. 2016 [40] | Y | Y | Y | Y | N | N | Y | Y |
| Xie et al. 2022 [41] | Y | Y | Y | Y | N | N | Y | Y |

**A2.** Appraisal of methodological quality of included cohort studies

| Citation | 1 | | 2 | 3 | 4 | 5 | 6 | 7 | 8 | 9 | 10 | 11 |
| --- | --- | --- | --- | --- | --- | --- | --- | --- | --- | --- | --- | --- |
| Grochulska et al. 2019 [42] | | Y | Y | Y | Y | Y | N | Y | Y | Y | N | Y |
| Kimata et al. 2014 [43] | | Y | Y | N | Y | Y | N | Y | Y | Y | N | Y |
| Narita et al. 2006 [2] | | Y | Y | Y | Y | Y | N | N | Y | Y | N | Y |
| Pisoni et al. 2006 [3] | | Y | Y | N | Y | Y | N | Y | Y | Y | N | Y |
| Plewig et al. 2019 [44] | | Y | Y | Y | N | N | N | Y | Y | Y | N | Y |
| Ramakrishnan et al. 2014 [45] | | Y | Y | Y | N | N | N | Y | Y | N | N | Y |
| Scherer et al. [46] | | Y | Y | Y | Y | Y | Y | Y | Y | Y | Y | Y |
| Sukul et al. 2021 [47] | | Y | Y | Y | N | N | N | Y | Y | N | N | Y |
| Sukul et al. 2023 [48] | | Y | Y | Y | Y | Y | Y | Y | Y | Y | Y | Y |

**A3.** Appraisal of methodological quality of included case-control study

| Citation | 1 | 2 | 3 | 4 | 5 | 6 | 7 | 8 | 9 | 10 |
| --- | --- | --- | --- | --- | --- | --- | --- | --- | --- | --- |
| Ibrahim et al. 2016 [50] | Y | N | N | Y | Y | N | N | Y | Y | Y |
